# Supplementary material for: Cell Line-Dependent Internalization, Persistence, and Immunomodulatory Effects of Staphylococcus aureus in Triple-Negative Breast Cancer
Source: Cancers (Basel). 2025 Sep 9;17(18):2947. doi: 10.3390/cancers17182947 (PMC12469023; doi:10.3390/cancers17182947)
Supplement: Supplementary file 1 [file cancers-17-02947-s001.zip › Supplementary file.pdf]

Figure S1: Optimization of gentamicin protection assay.

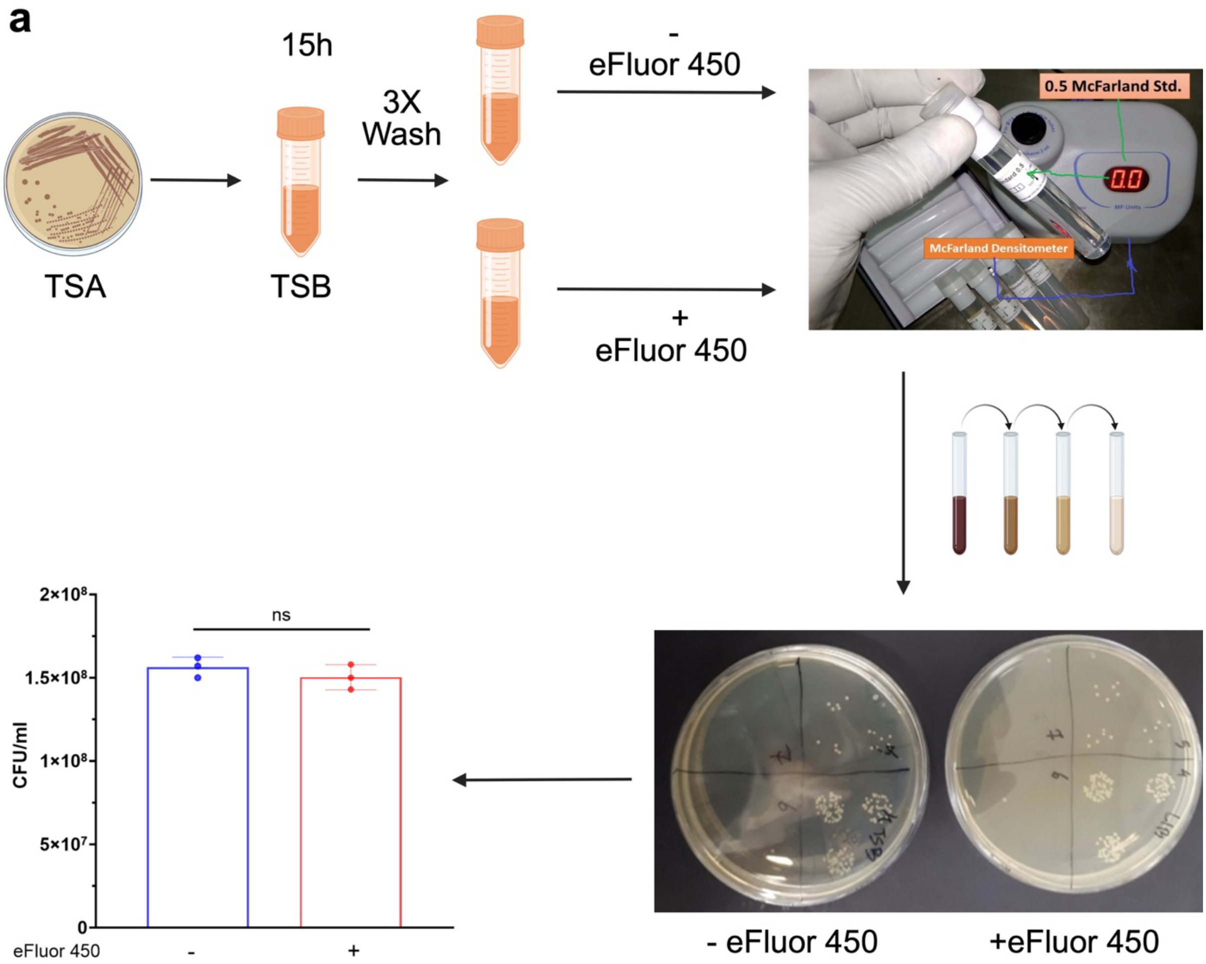

Figure S1a: Assessment of eFluor 450 labelling on *Staphylococcus aureus* viability. Colonies of *S. aureus* ATCC 25923 grown on Tryptone soya agar (TSA) were inoculated into Tryptone soya broth (TSB) and cultured for 15 hours at 37°C. The overnight cultures were then centrifuged at 3,200 × g for 10 minutes, washed three times with sterile, protein-free phosphate-buffered saline (PBS), and split equally between two tubes. The bacteria were resuspended in 750 µL of PBS with or without 10 µM eBioscience Cell Proliferation Dye eFluor 450 and incubated at 37°C in the dark for 30 minutes with gentle agitation. The bacteria were adjusted to 0.5 McFarland units (equivalent to 2×10^9 CFU), serially diluted, and plated on to TSA plates. Following overnight culture at 37°C the number CFU/mL were determined. Results are mean ± SD of three independent experiments. Labelled and unlabelled bacteria showed comparable viability (~1.5×10^8 CFU/mL), indicating no impact from the dye. Created in BioRender. Smith, E. (2025) https://BioRender.com/jpa333p.

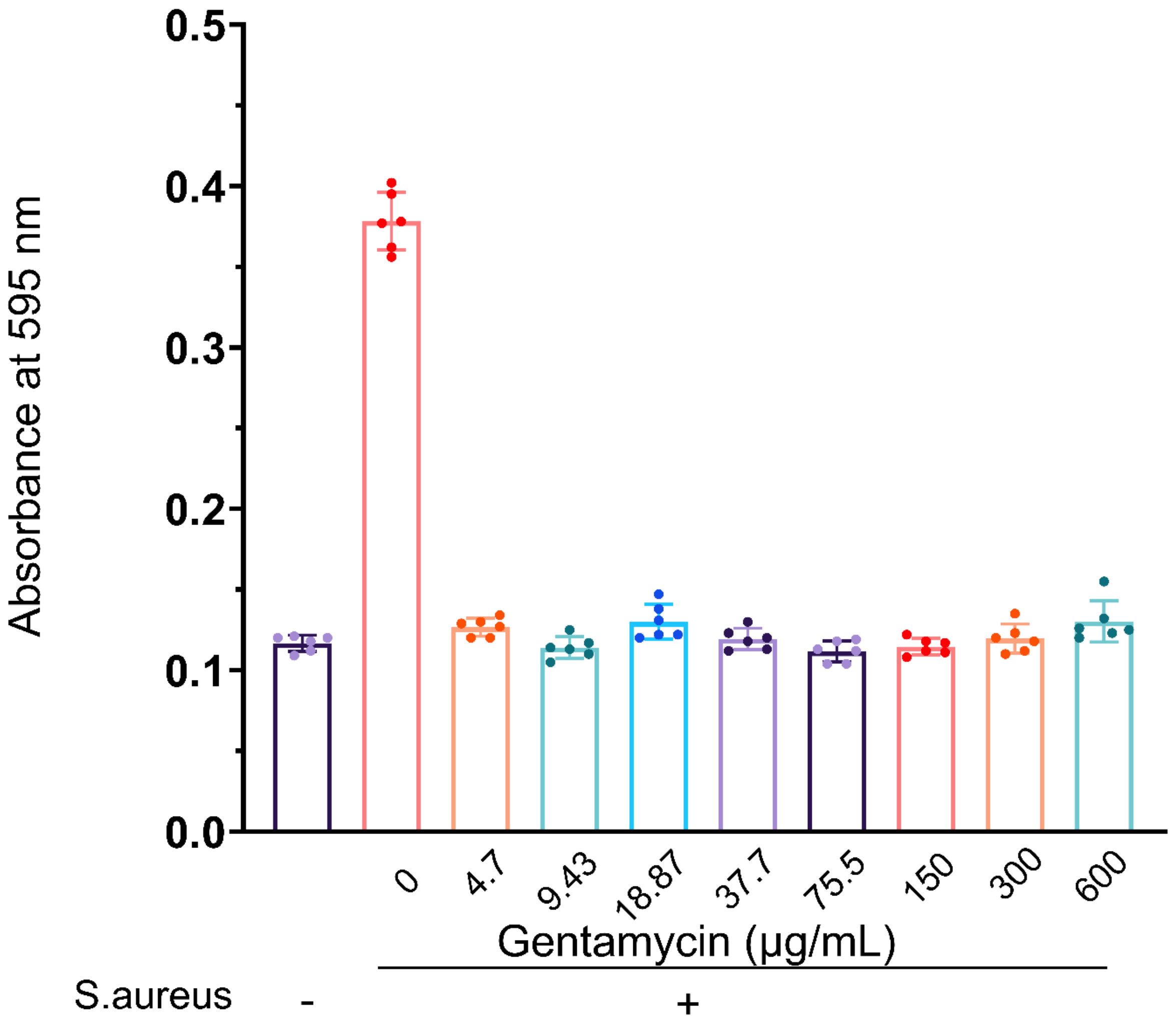

Figure S1b: Determination of *S. aureus* sensitivity to gentamicin using the minimum inhibitory concentration (MIC) assay. The MIC assay was performed according to established protocols [34] to assess *S. aureus* susceptibility to gentamicin. Compared to the 0 µg/mL control, no bacterial growth was observed across a concentration range of 4.5 to 600 µg/mL, indicating that *S. aureus* is broadly susceptible to gentamicin within this range.

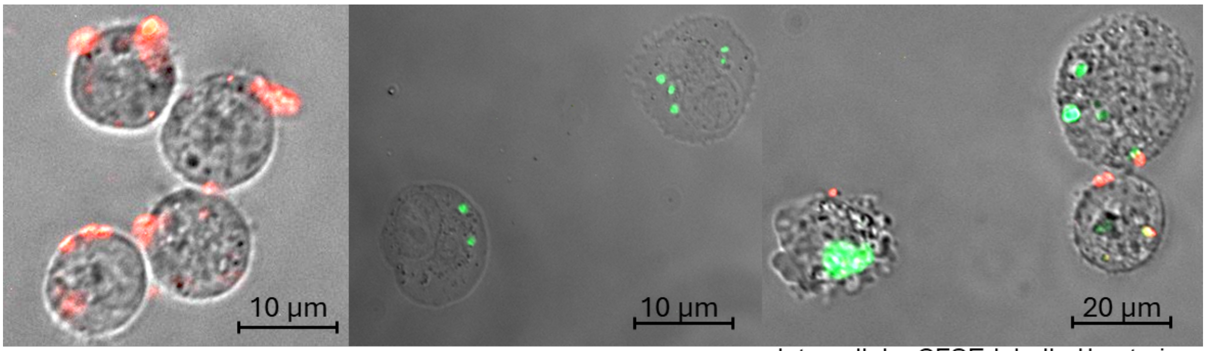

Dead extracellular bacteria stained red with PI

Intracellular CFSE-labelled bacteria stained green

Intracellular CFSE-labelled bacteria stained green and dead extracellular bacteria stained red with PI

Figure S1c: Confirmation of elimination of viable extracellular *S. aureus* using propidium iodide staining. *S. aureus* were labeled with carboxyfluorescein succinimidyl ester (CFSE) by incubating 1×10^9 CFU in 2 mL PBS with 10 µM CFSE (CellTrace CFSE Cell Proliferation Kit, Thermo Fisher Scientific, Cat# C34554). Breast cancer cell lines were seeded at 1.5×10^5 cells per well in 12-well plates and incubated overnight at 37°C with 5% CO_2. The following day, cells were washed with DPBS (Gibco, Cat# 14190144) and infected with CFSE-labeled *S. aureus* in DMEM supplemented with 10% FBS (antibiotic-free) at 37°C for 2 hours. To prevent photobleaching, infections were performed in the dark. Following infection, cells were washed three times with sterile DPBS and incubated with DMEM containing 10% FBS and 200 µg/mL gentamicin (Gibco, Cat# 15750060200) for 1 hour to eliminate extracellular bacteria. Cells were then stained with 5 µg/mL propidium iodide (PI) for 5 minutes and visualized by fluorescence microscopy. Dead extracellular bacteria stained red with PI, while viable intracellular bacteria retained green CFSE fluorescence, confirming effective elimination of extracellular bacteria and the presence of internalized *S. aureus*.

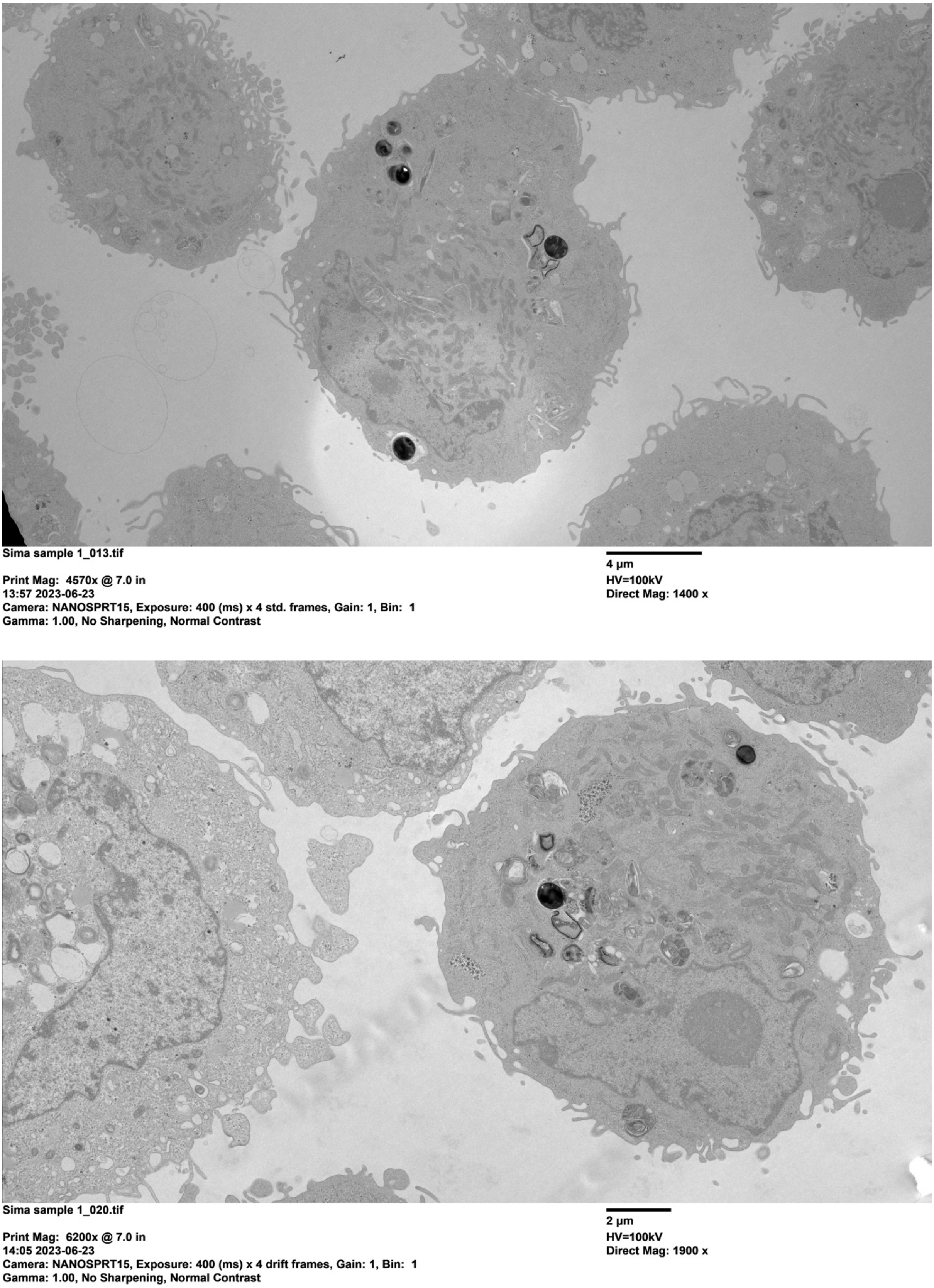

Figure S1d: Confirmation of intracellular *S. aureus* by transmission electron microscopy. Representative transmission electron microscopy images of MDA-MB-231 cells 24 hours post-infection, showing cells with and without intracellular *S. aureus*. Bacteria were observed within the cytoplasm and membrane-bound compartments, confirming successful internalization and intracellular localization.

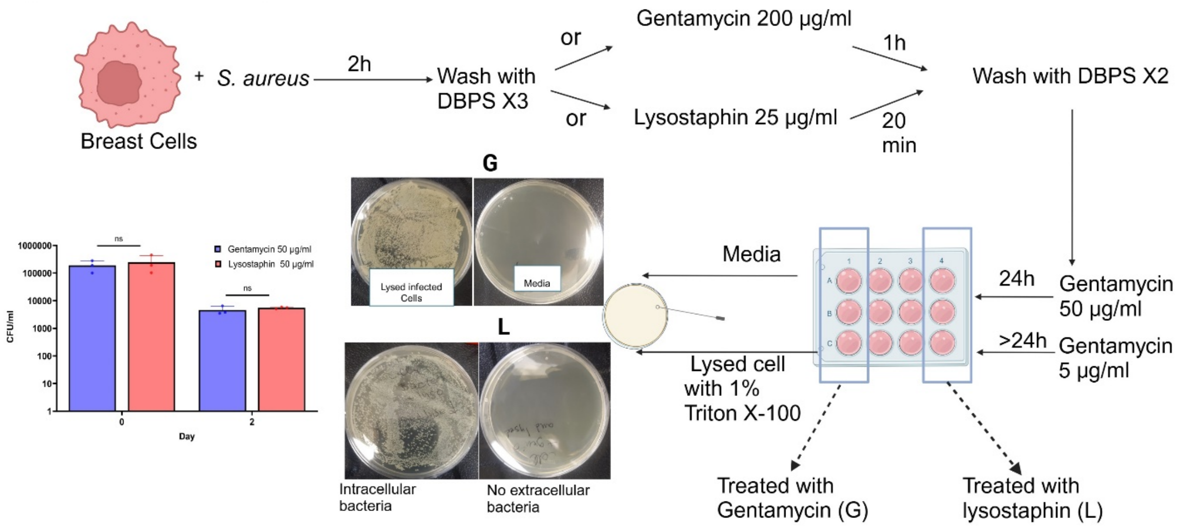

Figure S1e: Confirmation of viable intracellular *S. aureus* by CFU enumeration of lysed cell contents 24 hours post-infection. Breast cancer cell lines were seeded at 1.5 × 10^5 cells per well in 12-well plates and incubated overnight at 37°C with 5% CO_2. The following day, cells were washed with DPBS (Gibco, Cat# 14190144) and infected with eFluor 450-labeled *S. aureus* in antibiotic-free DMEM supplemented with 10% FBS for 2 hours at 37°C. To minimize photobleaching, infections were performed in the dark. Post-infection, cells were washed three times with sterile DPBS and treated with either 200 µg/mL gentamicin (Gibco, Cat# 15750060200) for 1 hour or 25 µg/mL lysostaphin (Sigma-Aldrich, Cat# 9011-93-2) for 20 minutes to eliminate extracellular bacteria. Cells were then washed twice with DPBS and incubated in DMEM containing 50 µg/mL gentamicin for up to 24 hours to maintain sterility. Following incubation, cells were centrifuged, and the culture medium was collected. Cells were lysed with 1% Triton X-100, and both cell lysates and collected medium were plated on TSA and incubated overnight for CFU enumeration. No bacterial growth was detected from the medium, confirming successful removal of extracellular bacteria. Comparable CFU/mL counts were observed between the two antibiotic treatment conditions at the time of infection and 24 hours post-infection, confirming the persistence of viable intracellular *S. aureus*. Data are presented as mean ± SEM from multiple independent experiments.

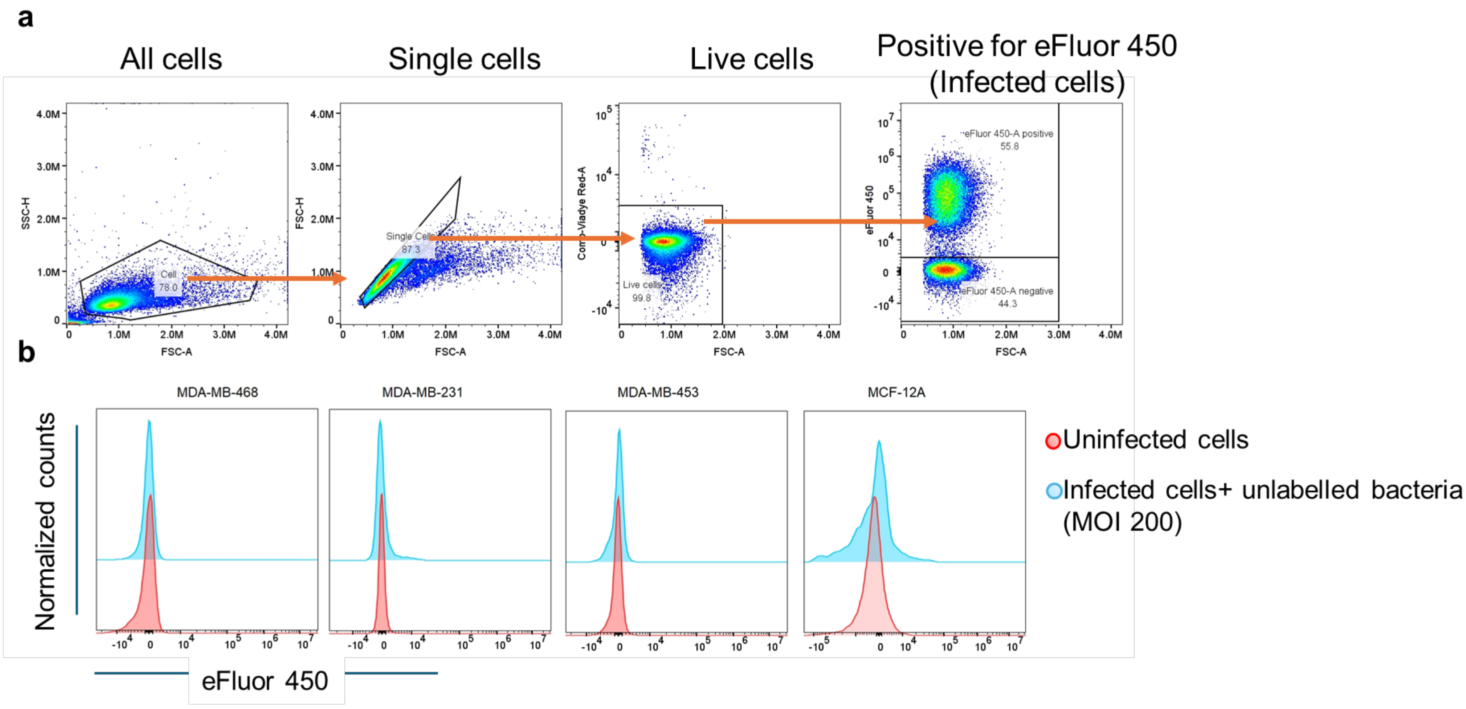

Figure S2: Spectral flow cytometry analysis of eFluor 450-labelled *S. aureus* in breast cell lines. (a) Gating strategy for identifying viable single cells and quantifying intracellular eFluor 450-labelled *S. aureus*. Cells were initially gated based on forward scatter (FSC) and side scatter (SSC) to exclude debris and free bacteria, followed by singlet gating to remove aggregates. Viable cells were identified using ViaDye Red Fixable Viability Dye, and *S. aureus*-positive cells were detected based on eFluor 450 fluorescence within the gated mammalian cell population. (b) Histogram overlays showing eFluor 450 fluorescence intensity in uninfected (red) and infected (MOI 200) (blue) breast cell lines (MDA-MB-468, MDA-MB-231, MDA-MB-453, and MCF-12A). No significant autofluorescence differences were observed between uninfected cells and those exposed to unlabelled *S. aureus*, confirming minimal background interference in the spectral flow cytometry analysis.

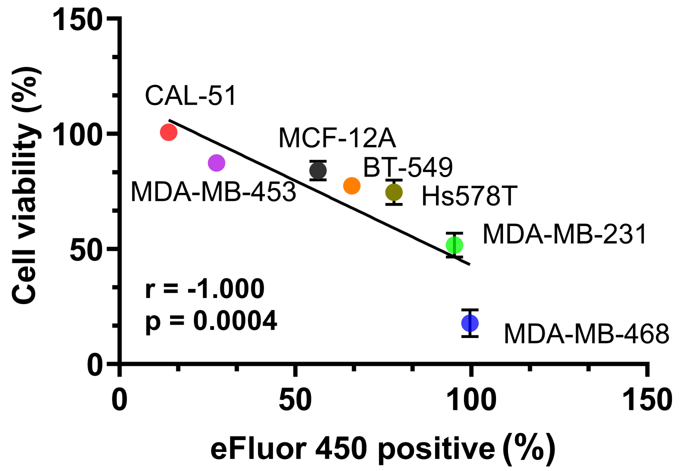

Figure S3: Correlation between bacterial internalization and cytotoxicity across different breast cell lines. Cells were infected with *Staphylococcus aureus* at a multiplicity of infection (MOI) of 200. The percentage of eFluor 450–positive cells (x-axis) indicate the level of bacterial internalization measured by flow cytometry. Cell viability (y-axis) was assessed 2 hours post-infection. A strong inverse correlation was observed between internalization and cell viability (Pearson's r = –1.000, p = 0.0004). Data represent the mean ± SD of n = 7 independent experiments.

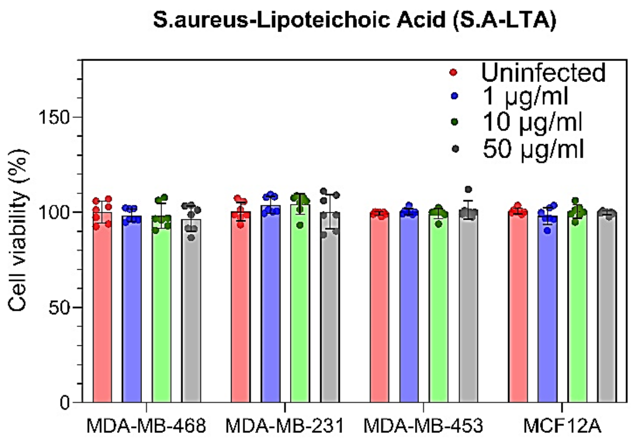

Figure S4: **Effect of LTA on cell proliferation.** Cells (MDA-MB-231, MDA-MB-468, MDA-MB-453 and MCF-12A) were seeded at 3.3 × 10^4 cell per well in 96-well plates and treated with LTA at 1, 10, and 50 µg/ml. After 5 days, cell proliferation was assessed using the crystal violet assay. LTA treatment at any concentration did not affect cell proliferation in any of the tested cell lines.

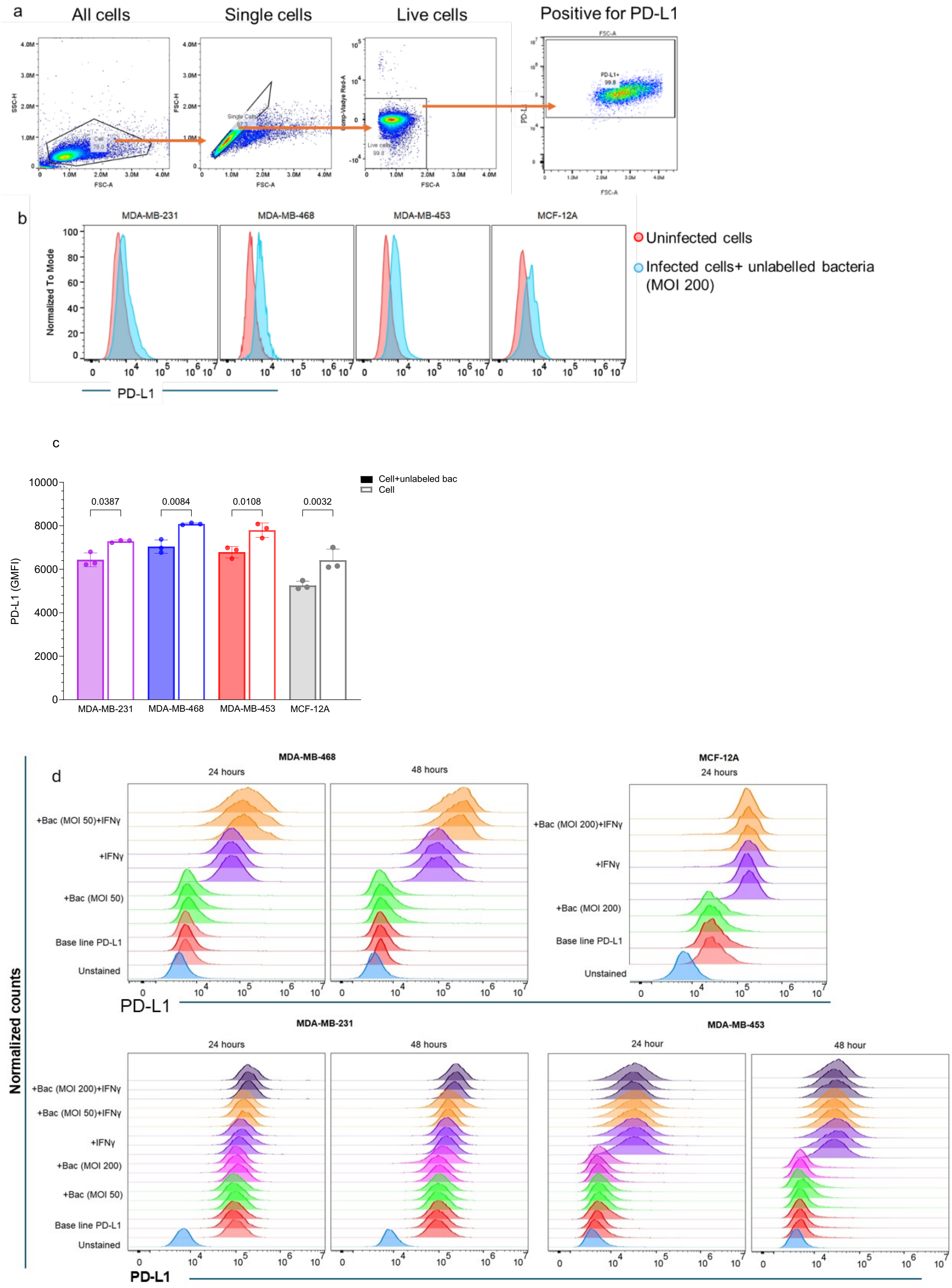
a
All cells
Single cells
Live cells
Positive for PD-L1
SSC-H
FSC-A
FSC-H
Single Cells
Comp-Viadye Red-A
Live cells
PD-L1
PD-L1+
99.8
b
MDA-MB-231
MDA-MB-468
MDA-MB-453
MCF-12A
Normalized To Mode
PD-L1
Uninfected cells
Infected cells+ unlabelled bacteria (MOI 200)
c
Cell+unlabeled bac
Cell
PD-L1 (GMFI)
0.0387
0.0084
0.0108
0.0032
MDA-MB-231
MDA-MB-468
MDA-MB-453
MCF-12A
d
MDA-MB-468
24 hours
48 hours
MCF-12A
24 hours
+Bac (MOI 50)+IFNγ
+IFNγ
+Bac (MOI 50)
Base line PD-L1
Unstained
+Bac (MOI 200)+IFNγ
+IFNγ
+Bac (MOI 200)
Base line PD-L1
Unstained
PD-L1
MDA-MB-231
24 hours
48 hours
MDA-MB-453
24 hour
48 hour
+Bac (MOI 200)+IFNγ
+Bac (MOI 50)+IFNγ
+IFNγ
+Bac (MOI 200)
+Bac (MOI 50)
Base line PD-L1
Unstained
Normalized counts
PD-L1

Figure S5: Spectral flow cytometry analysis of PD-L1 expression in breast cancer cells infected with S. aureus. Gating strategy for flow cytometry was used to detect PD-L1 expression using specific antibodies. All cells were first selected, followed by gating for single cells, then live cells, and finally identifying PD-L1-positive cells. (b) Flow cytometry histograms showing PD-L1 expression in uninfected cells (red) compared to cells infected with unlabelled S. aureus (blue) at MOI 50 for MDA-MB-468 and MOI 200 for the other cell lines.

(c) Quantification of PD-L1 geometric mean fluorescence intensity (GMFI) in uninfected cells and infected cells with unlabelled bacteria. A significant difference was observed between the two conditions, though the difference was not substantial. Background controls were established using a combination of uninfected cells and cells infected with unlabelled bacteria. (d) PD-L1 expression was measured in different breast cell lines infected with S. aureus at varying MOIs, with and without IFN-γ treatment, at 24- and 48-hours post-infection.

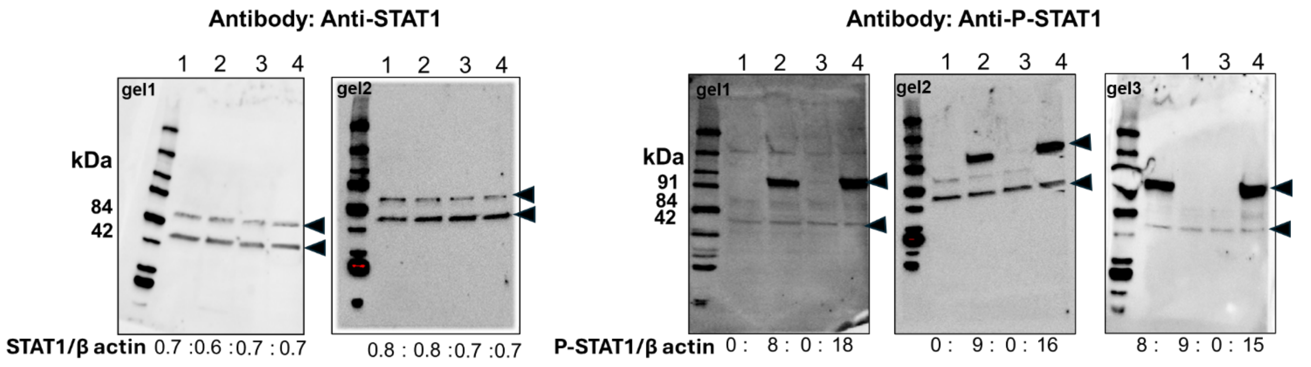

Figure S6: Uncropped Western blot images showing protein expression in MDA-MB-468 cells. Representative uncropped Western blots showing expression of STAT1 (84 kDa), phosphorylated STAT1 (p-STAT1, 91 kDa) and β-actin (42 kDa) in MDA-MB-468 cells. Treatments included infection with Staphylococcus aureus (MOI 50), IFN-γ (5 µM) for 24 hours, or their combination, as described in the main text. A total of 30–40 µg of total protein was loaded per lane. β-actin served as a loading control, and target protein levels were normalized to β-actin expression. Intensity ratios relative to β-actin are indicated for each band. Lane assignments: Lane 1 – Control, Lane 2 – IFN-γ (5 µM), Lane 3 – S. aureus, Lane 4 – S. aureus + IFN-γ (5 µM). Molecular weight markers are shown for reference.

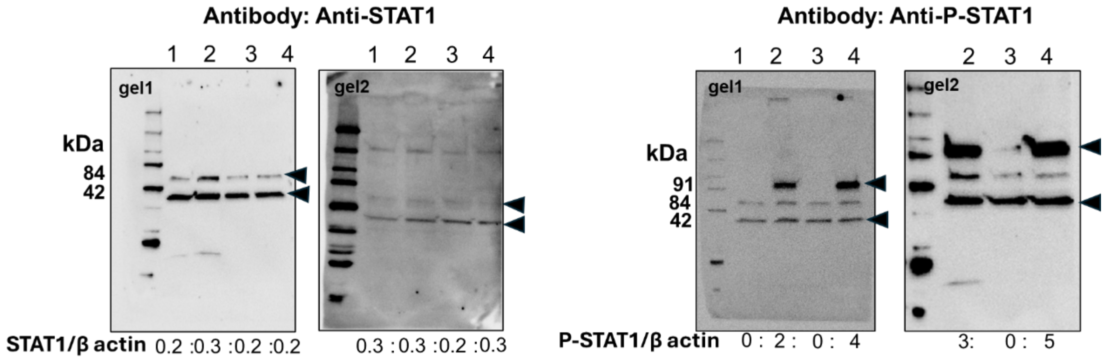

Figure S7: Uncropped Western blot images showing protein expression in MDA-MB-231 cells. Representative uncropped Western blots showing expression of STAT1 (84 kDa), phosphorylated STAT1 (p-STAT1, 91 kDa) and β-actin (42 kDa) in MDA-MB-231cells. Treatments included infection with Staphylococcus aureus (MOI 50), IFN-γ (5 µM) for 24 hours, or their combination, as described in the main text. A total of 30–40 µg of total protein was loaded per lane. β-actin served as a loading control, and target protein levels were normalized to β-actin expression. Intensity ratios relative to β-actin are indicated for each band. Lane assignments: Lane 1 – Control, Lane 2 – IFN-γ (5 µM), Lane 3 – S. aureus, Lane 4 – S. aureus + IFN-γ (5 µM). Molecular weight markers are shown for reference.

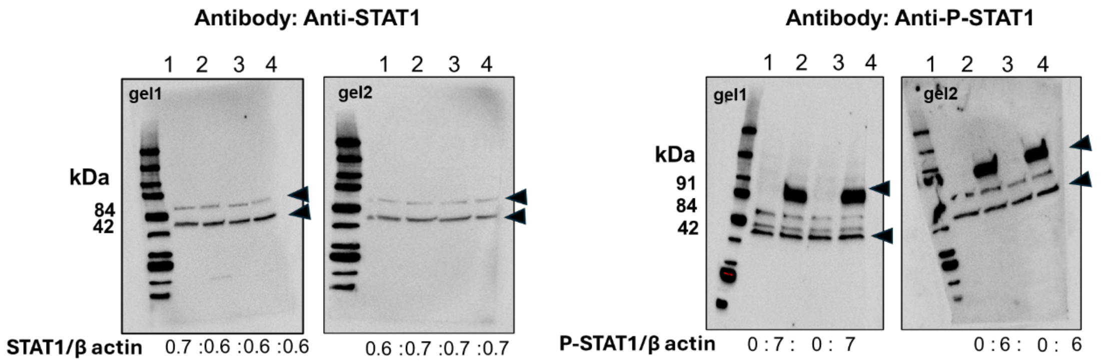

Figure S8: Uncropped Western blot images showing protein expression in MDA-MB-453 cells. Representative uncropped Western blots showing expression of STAT1 (84 kDa), phosphorylated STAT1 (p-STAT1, 91 kDa) and β-actin (42 kDa) in MDA-MB-453 cells. Treatments included infection with Staphylococcus aureus (MOI 50), IFN-γ (5 μM) for 24 hours, or their combination, as described in the main text. A total of 30–40 μg of total protein was loaded per lane. β-actin served as a loading control, and target protein levels were normalized to β-actin expression. Intensity ratios relative to β-actin are indicated for each band. Lane assignments: Lane 1 – Control, Lane 2 – IFN-γ (5 μM), Lane 3 – S. aureus, Lane 4 – S. aureus + IFN-γ (5 μM). Molecular weight markers are shown for reference.

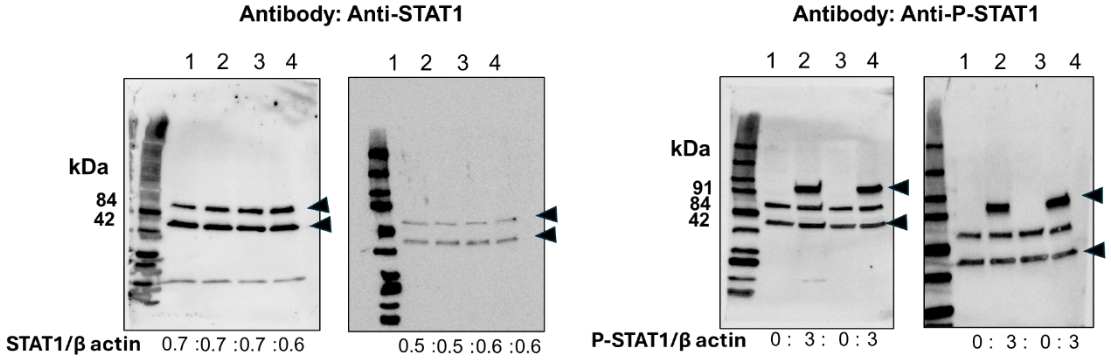

Figure S9: Uncropped Western blot images showing protein expression in MCF-12A cells. Representative uncropped Western blots showing expression of STAT1 (84 kDa), phosphorylated STAT1 (p-STAT1, 91 kDa) and β-actin (42 kDa) in MCF-12A cells. Treatments included infection with Staphylococcus aureus (MOI 50), IFN-γ (5 μM) for 24 hours, or their combination, as described in the main text. A total of 30–40 μg of total protein was loaded per lane. β-actin served as a loading control, and target protein levels were normalized to β-actin expression. Intensity ratios relative to β-actin are indicated for each band. Lane assignments: Lane 1 – Control, Lane 2 – IFN-γ (5 μM), Lane 3 – S. aureus, Lane 4 – S. aureus + IFN-γ (5 μM). Molecular weight markers are shown for reference.

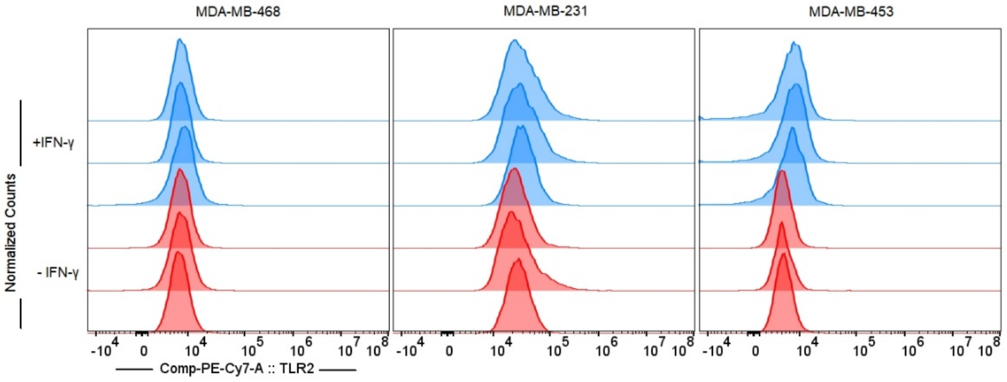

Figure S10: Spectral Flow Cytometry Analysis of TLR2 Expression in TNBC Cells Treated with IFN-γ. Cells were treated with 5 µM IFN-γ for 24 hours, and TLR2 expression was analyzed using spectral flow cytometry. IFN-γ treatment led to slightly increase in TLR2 expression in TNBC cells compared to baseline levels.

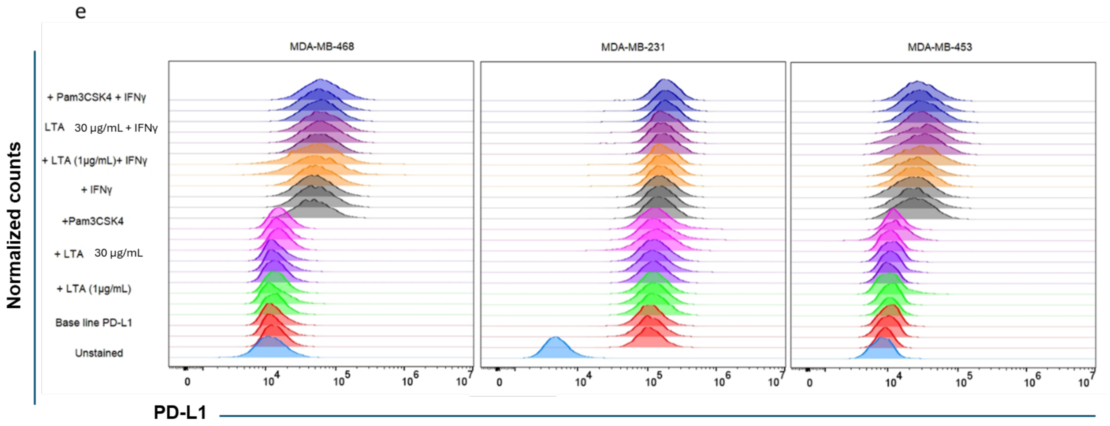

Figure S11: Spectral flow cytometry analysis of PD-L1 expression in breast cancer cells treated with TLR2 agonists. The gating strategy for flow cytometry to detect PD-L1 expression using specific antibodies was performed as outlined in Fig. S5. Briefly, all cells were initially selected, followed by sequential gating for single cells, live cells, and finally PD-L1–positive cells. PD-L1 expression was evaluated in TNBC cells treated with two TLR2 agonists (Pam3CSK4 at 1 µg/mL and S.A-LTA at 1 and 30 µg/mL) in the presence and absence of IFN-γ at 24 and 48 hours.
